# Hepatobiliary manifestations of COVID-19 and their impact on severity and outcomes in a single center in Saudi Arabia

**DOI:** 10.25122/jml-2022-0047

**Published:** 2022-08

**Authors:** Reem Al Argan, Mona Ismail, Dania AlKhafaji, Raed Alsulaiman, Fatimah Ismaeel, Reem AlSulaiman, Lameyaa Alsheekh, Tariq Alsaif, Feras Alkuwaiti, Abir Al Said, Safi Alqatari, Abrar Alwaheed, Alaa Alzaki, Marwan Al Wazzeh, Abdulaziz AlQuorain

**Affiliations:** 1 Department of Internal Medicine, College of Medicine, Imam Abdulrahman Bin Faisal University, Khobar, Saudi Arabia

**Keywords:** Coronavirus-2019, hepatic manifestations, severity, outcome, mortality

## Abstract

Recognizing hepatic manifestations of COVID-19 and their impact on the severity and outcome is crucial in managing this emerging pandemic. However, we lack such reported data in Saudi Arabia regarding this clinical entity. This is a retrospective observational study conducted on 387 patients with COVID-19 disease who were hospitalized at King Fahad Hospital of the University from March-September 2020. The total cohort was divided into two groups: liver and non-liver involvement. Then, the frequency of hepatic manifestations was determined, followed by comparing severity and outcome among the two study groups. A total of 387 patients were included, of which 72.87% had hepatic manifestations. The most prevalent abnormalities were high LDH in 308 (79.58%) followed by AST 205 (52.97%), GGTP 124 (31.26%), ALT 74 (19.12%), PT/INR 66 (17.05%), direct bilirubin 51 (12.40%), total bilirubin 46 (11.88%), and low albumin 48 (12.4%). Univariate analyses showed that liver involvement was significantly associated with severe (31.91%) and critical (34.75%) presentation (P<0.001). Multivariate regression analysis showed that the presence of liver involvement was an independent risk factor for severe or critical COVID-19 disease (OR 2.44; P<0.001), longer hospitalization (OR 2.27; P=0.001), and ICU admission (OR 2.27; P=0.006). The current study showed that liver involvement is common in the setting of COVID-19 disease. Such patients had a higher disease severity and a worse clinical outcome.

## INTRODUCTION

World Health Organization (WHO) announced Coronavirus disease 2019 (COVID-19) as a pandemic in March 2020 [1]. There were 250 million cases infected globally by COVID-19, with 5 million deaths as of 15^th^ November 2021 [2]. Saudi Arabia is highly affected by COVID-19 disease. Since the beginning of the pandemic, 549,000 confirmed cases had been reported to WHO, with almost 8,800 deaths at the time of writing this report [3]. The earliest reports from China reported respiratory symptoms as the most common presentation of COVID-19, primarily cough, fatigue, and shortness of breath [4]. This could progress into acute respiratory distress syndrome (ARDS) and lead to the need for intensive care unit (ICU) admission and mechanical ventilation [4]. However, extra pulmonary manifestations of COVID-19 are currently well recognized, such as cardiac, gastrointestinal, hepatic, and hematological manifestations [5].

Hepatobiliary manifestations of COVID-19 disease are common, being reported in 21–53% of COVID-19 cases [4–6]. High bilirubin, alanine aminotransferase (ALT), and aspartate aminotransferase (AST) levels were the most commonly reported manifestations [4–6]. Several studies found a link between hepatic dysfunction and the severity of COVID-19 disease [4, 5]. Guan *et al*. noticed that liver derangement was greater in patients with severe disease [4]. Additionally, Huang *et al*. reported a higher percentage of liver enzyme elevation in ICU patients [5]. The evidence about the impact of hepatic dysfunction on the outcome of COVID-19 is heterogeneous. For instance, a retrospective case series of 113 deceased cases found that the concentration of liver enzymes was higher in deceased than recovered patients [7]. However, According to Yang *et al*., there was no difference in hepatic dysfunction between COVID-19 survivors and non-survivors [8].

Recognizing COVID-19 hepatic manifestations and their impact on the severity and outcome is crucial in managing such a pandemic. However, we lack such evidence in the Middle East. Therefore, we believe that our study could contribute to a better understanding of such an important and common manifestation of COVID-19 in the region. Furthermore, we expect that this study will help clinicians to manage their patients accordingly and, as a result, improve their outcomes.

## Material and Methods

### Data Collection

This is a retrospective observational study of patients hospitalized with COVID-19 at King Fahd Hospital of the Imam Abdulrahman Bin Faisal University, Eastern Province, Saudi Arabia, from March-September 2020. A total of 387 patients were included in the study. Exclusion criteria included cancer, immunodeficiency disorders, human immunodeficiency virus infection, and pregnancy.

The following data was divided into six sections. The first section included baseline data (age, gender, nationality, and comorbid conditions). The second section represented baseline laboratory investigations, including white blood cell (WBC) count with lymphocyte and neutrophils count, haemoglobin, platelets, creatinine, C-reactive protein (CRP), erythrocyte sedimentation rate (ESR), ferritin and D-dimer. The third section included the liver profile data: (total bilirubin, direct bilirubin, aspartate aminotransferase (AST), alanine aminotransferase (ALT), gamma-glutamyl transpeptidase (GGTP), alkaline phosphatase (ALP), and lactate dehydrogenase (LDH)). The fourth section included data on synthetic liver function (albumin level and prothrombin time (PT)/international normalized ratio (INR)). Liver enzyme abnormalities were graded based on the followings: mild: less than 2 times above the upper limit of normal, moderate: 2–5 times the upper limit of normal, and severe: more than 5 times the upper limit of normal. Albumin level was graded based on the followings: mild: 3.0–3.5 g/dl, moderate: 2.9–2.0 g/dl, severe: less than 2.0 g/dl. PT/INR level was graded based on the following: mild: less than 1.7, moderate: 1.7–2.3, severe: more than 2.3. The fifth section included the severity of COVID-19, data classified according to the guidelines published by the Saudi Arabian Ministry of Health in 2020 [9]: A) mild-moderate: negative pneumonia on chest X-ray and no oxygen requirement. B) severe disease: oxygen saturation <93% on room air, respiratory rate ≥30/minute, partial pressure of oxygen/fraction of inspired oxygen<300 or lung infiltrates >50% of the lung field within 24–48 hours. C) critical disease can be classified if any of the following is present: acute respiratory distress syndrome (ARDS), sepsis, altered level of consciousness, multi-organ failure, or with risk factors of cytokine storm syndrome defined as ferritin more than 600 ug/L at initial presentation and LDH more than 250 U/L or high D-Dimer more than 1 mcg/ml. The final section was about the outcome of COVID-19 disease, which was defined by the length of hospital stay, the requirement for ICU admission, mechanical ventilation, and death.

Then, the frequency of hepatobiliary manifestations was determined. After that, we divided the total population into two groups: the first one was the group with liver involvement, defined as any elevation of total bilirubin, direct bilirubin, ALT, AST, ALP, GGTP, PT/INR above the upper limit of reference range or low albumin below the lower limit of the reference range. High LDH was not included as a parameter of liver involvement since it can be caused by COVID-19 itself and not necessarily liver involvement [10]. The second group is the one without liver involvement. After that, the severity and outcome of both groups were investigated.

### Statistical analysis

Means and standard deviations (SD) or medians and inter-quartile ranges (IQR) were used to report continuous data, while absolute numbers and percentages were used to report categorical data. Groups were compared using student t-test, Mann Whitney u test for continuous data, Chi-square test, or Fisher's exact test for categorical data. Multiple logistic regression was used to study the multivariate association between study outcome and risk factors. All statistical analyses were done using SAS version 9.2 (SAS Institute, Inc, Cary, NC) and (R Foundation for Statistical Computing, Vienna, Austria). A significant P value was set at less than 0.05.

## Results

### Demographics and comorbidities

A total of 387 patients were included. The median age was 52.18±15.66 years. 104 (26.87%) were females, and 283 (73.13%) were males. 214 (55.30%) had Saudi nationality, and 173 (44.70%) were non-Saudis. Diabetes mellitus (DM) was the most prevalent comorbidity in 184 (47.55%), followed by hypertension in 148 (38.24%). Pre-existing liver diseases were found in 8 patients (2.07%) (Table 1). Liver involvement was found in 282 cases (72.87%). A comparison of age and comorbidities between the two study groups showed that the majority of the liver involvement group were males (79.79% *vs*. 55.24%; P<0.001) and of non-Saudi nationality (50% *vs*. 30.48%; P<0.001). Although it did not reach statistical significance, they tended to be older (53.18±15.00 *vs*. 50.23±16.81).

**Table 1:** Demographics and comorbidities.

	Liver involvement N=282 (72.87%)	No Liver involvement N=105 (27.13%)	Total N=387	P-value
**Age**	53.18±15.00	50.23±16.81	52.18±15.66	0.096
**Gender**				
Female	57 (20.21%)	47 (44.76%)	104 (26.87%)	**<0.001**
Male	225 (79.79%)	58 (55.24%)	283 (73.13%)	
**Nationality**				
Saudi	141 (50.00%)	73 (69.52%)	214 (55.30%)	**<0.001**
Non-Saudi	141 (50.00%)	32 (30.48%)	173 (44.70%)	
**Diabetes Mellitus**	128 (45.39%)	56 (53.33%)	184 (47.55%)	0.164
**Hypertension**	109 (38.65%)	39 (37.14%)	148 (38.24%)	0.786
**Chronic Respiratory Diseases**	14 (4.96%)	8 (7.62%)	22 (5.68%)	0.316
**Chronic Kidney Diseases**	10 (3.55%)	8 (7.62%)	18 (4.65%)	0.091
**Gastrointestinal Diseases**	9 (3.19%)	3 (2.86%)	11 (2.84%)	0.45
**Chronic liver diseases**	6 (2.13%)	2 (1.9%)	8 (2.07%)	0.015

N – Number. Significant P-value is shown in bold.

### Description of liver enzymes abnormalities

The most prevalent liver enzyme abnormality was high LDH in 308 (79.58%) followed by AST 205 (52.97%), GGTP 121 (31.26%), ALT 74 (19.12%), direct bilirubin 51 (12.40%) and total bilirubin 49 (11.88%). ALP elevation was the least prevalent in 26 (6.7%). Low albumin was found in 48 (12.40%), with high PT/INR in 66 (17.05%). Liver enzymes elevation was commonly mildly elevated, followed by moderate elevation, and least commonly severely elevated (Table 2).

**Table 2:** Descriptive analysis of liver abnormalities.

Parameter	Normal range	Frequency (Percentage)	Median (IQR)	Severity, Frequency (Percentage)		
				Mild	Moderate	Severe
**Total Bilirubin**	(0.2–1.2 mg/dl)	46 (11.88%)	0.60 (0.50)	40 (10.34%)	6 (1.55%)	NA
**Direct Bilirubin**	(0.1–0.5 mg/dl)	51 (12.40%)	0.30 (0.20)	38 (9.82%)	8 (2.07%)	2 (0.52%)
**ALT**	(7–55 U/L)	74 (19.12%)	29.00 (26.00)	56 (14.47%)	15 (3.88%)	3 (0.78%)
**AST**	(5–34 U/L)	205 (52.97%)	36.00 (35.00)	127 (32.82%)	71 (18.35%)	7 (1.81%)
**ALP**	(40–150 U/L)	26 (6.71%)	71.00 (38.00)	20 (5.17%)	5 (1.29%)	1 (0.26%)
**GGTP**	(12–64 U/L)	121 (31.26%)	43.00 (51.00)	78 (20.16%)	36 (9.30%)	7 (1.81%)
**LDH**	(81–234 U/L)	308 (79.58%)	358.0 (259.0)	189 (48.84%)	109 (28.17%)	10 (2.58%)
**Albumin**	(3.2–5.2 g/dl)	48 (12.40%)	3.70 (0.70)	26 (6.74%)	16 (4.15%)	1 (0.26%)
**PT/INR**	≤1.1	66 (17.05%)	1.00 (0.15)	47 (14.51%)	3 (0.93%)	16 (4.94%)

IQR – Interquartile range; NA – Not applicable; ALT – Alanine aminotransferase; AST – Aspartate aminotransferase; ALP – Alkaline phosphatase; GGTP – Gamma-glutamyl transpeptidase; LDH – Lactate dehydrogenase; PT/INR – Prothrombin time/International normalized ratio.

### Comparison of laboratory abnormalities between the two study groups

Patients with liver involvement had higher median neutrophil count (5.0 *vs*. 3.8; P=0.003), CRP (8.4 *vs*. 3.00; P<0.001), D-dimer (0.95 *vs*. 0.53; P<0.001) and ferritin (618 *vs*. 240.7; P<0.001), ESR tended to be higher in the liver involvement group, but it was statistically insignificant. In addition, the same group had lower median lymphocyte count (1.2 *vs*. 1.4; P=0.016) and platelets (199 *vs*. 228; P=0.002) (Table 3).

**Table 3:** Comparison of laboratory values between the liver and non-liver involvement groups.

Laboratory Test	Normal Range	Liver involvement Median (IQR)	No Liver involvement Median (IQR)	Total Median (IQR)	P-value
WBC	(4.0–11 k/ul)	7.00 (5.30)	6.00 (4.10)	6.70 (5.00)	0.154
Haemoglobin	Males (13.0–18.0 g/dl) Females (12.0–16.0 g/dl)	13.30 (2.60)	13.00 (2.40)	13.20 (2.70)	0.144
Platelets	(140–450 k/ul)	199.0 (105.0)	228.0 (91.00)	209.0 (104.0)	**0.002**
Neutrophils	(2.0–7.5 k/ul)	5.00 (4.60)	3.80 (2.90)	4.70 (4.10)	**0.003**
Lymphocytes	(1.0–5.0 k/ul)	1.20 (0.87)	1.40 (0.75)	1.20 (0.80)	**0.016**
Creatinine	(0.6–1.2 mg/dl)	0.95 (0.42)	0.90 (0.37)	0.94 (0.41)	0.174
ESR	(0–20 mm/hour)	44.00 (35.00)	39.00 (44.00)	43.00 (37.00)	0.085
CRP	(0.1–0.5 mg/dl)	8.40 (10.78)	3.00 (8.82)	7.40 (11.22)	**<0.001**
D-dimer	(≤0.5 ug/ml)	0.95 (1.38)	0.53 (0.83)	0.85 (1.24)	**<0.001**
Ferritin	(21.81–274.66 ng/ml)	618.0 (1072)	240.7 (396.9)	524.0 (814.7)	**<0.001**

IQR – interquartile range; WBCs – White blood cells; ESR – Erythrocyte sedimentation rate; CRP – C-reactive protein. Significant P-value is shown in bold.

### Association of liver involvement with the severity and outcome of COVID-19

**Figure 1:**
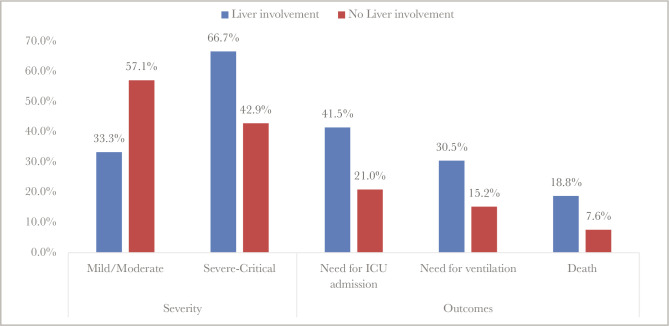
Comparison of severity and outcome between the two study groups (univariate analysis).

Univariate analysis was done to compare the severity of COVID-19 pneumonia between the two groups. The group of liver involvement was significantly associated with a severe and critical presentation in 31.91 and 34.75% of cases, respectively (P<0.001). In addition, we found a significant association with all the severe and critical MOH severity criteria (P<0.05) (Table 4, Figure 1). Moreover, the group with liver involvement had a longer hospital stay, higher requirement for ICU admission, mechanical ventilation, and death (P<0.05) (Table 4, Figure 1).

**Table 4:** Comparison of severity and outcome between the two study groups (univariate analysis).

	Liver involvement	No Liver involvement	Total	P-value
**MOH Severity Criteria**				
Mild/Moderate	94 (33.33%)	60 (57.14%)	154 (39.79%)	**<0.001**
Severe	90 (31.91%)	30 (28.57%)	120 (31.01%)	
Critical	98 (34.75%)	15 (14.29%)	113 (29.20%)	
**Severe**				
Pneumonia on chest x ray	231 (82.21%)	66 (62.86%)	297 (76.94%)	**<0.001**
Respiratory rate greater than 30/minute	126 (44.68%)	22 (20.95%)	148 (38.24%)	**<0.001**
Oxygen saturation less than 93% on room air	192 (68.09%)	43 (40.95%)	235 (60.72%)	**<0.001**
PaO2/FiO2 ratio less than 300	127 (48.29%)	22 (23.16%)	149 (41.62%)	**<0.001**
Lung infiltration greater than 50% of lung fields within 24–48 hours	118 (41.84%)	19 (18.10%)	137 (35.40%)	**<0.001**
**Critical**				
Adult Respiratory distress syndrome	91 (32.27%)	18 (17.14%)	109 (28.17%)	**0.003**
Sepsis	60 (21.28%)	7 (6.67%)	67 (17.31%)	**<0.001**
Altered mental status	54 (19.15%)	8 (7.62%)	62 (16.02%)	**0.006**
Multiorgan failure	47 (16.67%)	5 (4.76%)	52 (13.44%)	**0.002**
Cytokine storm syndrome	102 (37.78%)	10 (9.80%)	112 (30.11%)	**<0.001**
**Outcome**				
Length of hospital stay	14.79±16.17	10.51±12.12	13.54±15.25	**0.014**
Need for ICU admission	117 (41.49%)	22 (20.95%)	139 (35.92%)	**<0.001**
Need for ventilation	86 (30.50%)	16 (15.24%)	102 (26.36%)	**0.002**
Death	53 (18.79%)	8 (7.62%)	61 (15.76%)	**0.007**

PaO2/FiO2 – Partial pressure of Oxygen/Fraction of inspired oxygen. Significant P-value is shown in bold.

Multivariate analysis showed that age (OR 1.05; P<0.001), non-Saudi nationality (OR 1.86; P<0.017), and DM (OR 1.76; P<0.037) were independent risk factors for severe or critical COVID-19 pneumonia. It also confirmed that liver involvement increased the odds of severe or critical COVID-19 disease by 2.44 times (P<0.001) (Table 5). In addition, the same analysis was done to examine the association with the outcome variables. There was a statistically significant association between older age and longer hospitalization (OR 1.02; P=0.009), a higher need for ICU admission (OR 1.04; P<0.001), mechanical ventilation (OR 1.05; P<0.001), and death (OR 1.07; P<0.001). In addition, non-Saudi nationality was associated with a significantly higher need of ICU admission (OR 2.09; P=0.005), mechanical ventilation (OR 2.67; P<0.001) and death (OR 3.46; P<0.001). Moreover, liver involvement increased the odds of prolonged hospitalization and ICU admission by 2.27 times (P=0.001 and 0.006, respectively). Although the liver involvement group had a higher likelihood of mechanical ventilation and death, the difference was statistically insignificant (Table 6).

**Table 5:** Logistic regression of severity of COVID-19 disease with demographics and liver involvement.

Covariate	Severe or critical OR (95% CI)	P-Value
Age	1.05 (1.034–1.08)	**<0.001**
Gender (Male)	1.23 (0.698–2.16)	0.476
Nationality (Non-Saudi)	1.86 (1.116–3.12)	**0.017**
Diabetes Mellitus	1.76 (1.035–3.00)	**0.037**
Hypertension	1.00 (0.549–1.82)	0.999
Respiratory Diseases	0.79 (0.281–2.21)	0.650
Chronic kidney diseases	1.86 (0.583–5.92)	0.295
Pre-existing GI and liver diseases	1.60 (0.523–4.89)	0.410
Liver Involvement	2.44 (1.432–4.16)	**<0.001**

OR – Odds ratio; CI – Confidence interval; GI – gastrointestinal. Significant P-value is shown in bold.

**Table 6:** Logistic regression of association of outcome of COVID-19 disease with demographics and liver involvement.

Covariate	Outcome							
	*Length of hospitalization OR (95% CI)	P-value	ICU admission OR (95% CI)	P-value	Mechanical ventilation OR (95% CI)	P-value	Death OR (95% CI)	P-value
**Age**	1.02 (1.006–1.04)	**0.009**	1.04 (1.023–1.06)	**<0.001**	1.05 (1.025–1.07)	**<0.001**	1.07 (1.043–1.10)	**<0.001**
**Gender**	1.16 (0.681–1.96)	0.589	1.41 (0.778–2.55)	0.258	1.99 (0.998–3.98)	0.051	2.13 (0.872–5.21)	0.097
**Nationality**	1.59 (0.981–2.57)	0.060	2.09 (1.256–3.47)	**0.005**	2.67 (1.52–4.70)	**<0.001**	3.46 (1.661–7.20)	**<0.001**
**Diabetes mellitus**	1.39 (0.844–2.30)	0.194	1.38 (0.832–2.29)	0.212	1.57 (0.906–2.71)	0.108	1.48 (0.761–2.90)	0.247
**Hypertension**	1.19 (0.673–2.09)	0.555	1.17 (0.671–2.05)	0.579	1.10 (0.603–1.99)	0.765	1.10 (0.542–2.23)	0.792
**Respiratory diseases**	0.71 (0.27–1.87)	0.487	1.32 (0.481–3.64)	0.588	1.35 (0.457–3.99)	0.587	1.96 (0.579–6.64)	0.279
**Chronic kidney diseases**	2.68 (0.814–8.84)	0.105	2.13 (0.745–6.12)	0.158	1.35 (0.441–4.13)	0.599	2.25 (0.652–7.76)	0.199
**Pre-existing GI and liver diseases**	1.97 (0.664–5.83)	0.222	1.75 (0.597–5.11)	0.308	1.93 (0.614–6.06)	0.261	3.08 (0.824–11.53)	0.094
**Liver involvement**	2.27 (1.379–3.74)	**0.001**	2.27 (1.264–4.07)	**0.006**	1.83 (0.949–3.52)	0.071	2.37 (0.966–5.82)	0.060

OR – Odds ratio; CI – Confidence interval; GI – gastrointestinal. *Length of hospitalization: (we used a cut-off of more than 7 days) correlated with the reported median duration of hospital stay in Saudi Arabia. Significant P-value is shown in bold.

## Discussion

This is an observational study of 387 hospitalized COVID-19 patients describing hepatic manifestations associated with COVID-19 disease and examining their impact on the severity and outcome of the disease. Our study shows the following key findings: first, 72.87% of our cohort had liver involvement, most commonly high LDH, followed by AST, GGTP, ALT, PT/INR, bilirubin, and low albumin. Second, the group with liver involvement had greater neutrophil, CRP, D-dimer, and ferritin levels but lower lymphocyte and platelet counts. Following that, the presence of liver involvement increased the odds of severe or critical COVID-19 disease. Furthermore, it was an independent risk factor for prolonged hospitalization and ICU admission. Finally, older age and non-Saudi nationality were linked to severe or critical COVID-19 pneumonia, as well as poor outcomes. DM increased the likelihood of severe or critical COVID-19 infection.

Our study is in line with a previous report of 417 COVID-19 patients that looked at the frequency of liver involvement secondary to COVID-19 disease. They found that 76.3% of their population had an abnormal liver profile [11]. However, earlier studies reported a lower prevalence, ranging between 14 and 54% at the beginning of the pandemic [12]. A meta-analysis by Wu *et al*. of 45 studies showed that the pooled incidence of any abnormal liver enzyme was 27.2% at admission and 36% during hospitalization [13]. Looking into the prevalence of individual liver enzyme abnormalities, our study shows that high LDH was the most prevalent abnormality in 79.58%, followed by AST at 52.97%, GGTP at 31.26%, ALT at 19.12%, direct bilirubin at 12.40%, total bilirubin 11.88%, and least commonly ALP elevation in 6.71%. We must look at LDH very carefully because it could be secondary to COVID-19 itself and not necessarily liver injury [10]. Therefore, we excluded high LDH from the definition of the liver involvement group. Most other studies looked specifically at ALT and AST elevation. For example, a study of 69 patients treated for SARS-CoV2 infection in early 2020 reported an increase in ALT and AST in 33% and 28% of patients, respectively [14]. Guan *et al*. evaluated 1099 COVID-19 patients and showed that 22.2% had elevated AST and 21.3% had elevated ALT. Furthermore, in a research of 5700 individuals from New York, USA, the prevalence of raised AST and ALT was shown to be greater, at 58.4% and 39.0%, respectively [15]. Another study was conducted in China on 2,922 patients and found that 48.6% of COVID-19 patients developed abnormal liver profiles: high ALT in 22.7%, AST in 7.6%, ALP in 4.6%, and GGTP in 18.5% [16]. However, Wu *et al*. reported abnormal albumin as the most common liver manifestation in patients with COVID-19, followed by GGTP, AST, ALT, total bilirubin, and ALP [13]. Our findings showed that liver enzyme elevation was commonly mildly elevated, consistent with evidence showing that ALT and AST levels were commonly elevated below 100 U/L [14].

According to our findings, the group with liver involvement had higher neutrophil, CRP, D-dimer, and ferritin levels with lower lymphocyte and platelet counts. Similarly, Fan *et al*. reported that patients with abnormal liver profiles had higher procalcitonin (P=0.0001) and CRP levels (P=0.0105) [17]. In addition, Zhang *et al*. found higher levels of WBCs, neutrophils, CRP, and D-dimer in patients with abnormal liver profiles secondary to COVID-19 disease (P<0.05) [18]. Another study found that the levels of cytokines such as interleukins were more elevated in patients with high ALT than patients with normal ALT (P<0.05) [19].

In our study, 31.91% and 34.75% of patients with liver involvement had severe-critical COVID-19 pneumonia, respectively (P=0.001). Regression analysis showed that liver involvement increases the likelihood of severe or critical COVID-19 infection by 2.44 times (P<0.001). Similarly, previous studies have linked liver injury to severe COVID-19 infection [4, 20]. The same study by Guan *et al*. reported that patients with severe diseases had higher transaminitis than the non-severe disease (ALT 28.1% *vs*. 19.8%, AST 39.4% *vs*. 18.2%) [4]. Similar results were also confirmed in a meta-analysis of 20 retrospective studies of 3428 patients with SARS-COV2 infection, where 1455 patients had severe disease [21]. They also had statistically higher AST, ALT, and total bilirubin [21]. Huang *et al*. found that high liver enzymes (AST, ALT, GGTP, and LDH) were substantially linked with the severity of COVID-19 disease from baseline to 30 days after admission in a retrospective cohort analysis of 1003 COVID-19 hospitalized patients in China (P=0.05) [22]. The same meta-analysis by Wu *et al*. found that severe and/or critical COVID-19 patients had a significantly higher pooled incidence of abnormal liver biochemical indicators at admission than mild and/or moderate patients [13]. Furthermore, Cai *et al*. found that the prevalence of abnormal liver tests and liver injury in the Chinese population was linked to the progression to severe COVID-19 infection [11]. On the other hand, some studies reported no significant variation in ALT levels of COVID-19 patients based on disease severity [23].

In our report, multivariate analysis proved that liver involvement increased hospitalization length and ICU admission odds by 2.27 times (P<0.05). Similarly, in January of 2020, a study from Wuhan, China, showed that AST was elevated in 62% of ICU patients *vs*. 25% of non-ICU patients [4]. In addition, another study found that the mean hospital stay was longer in COVID-19 patients with impaired liver function (15.09±4.79 days) than in patients with normal liver function (12.76±4.14 days) (P=0.021) [17]. Moreover, Yip *et al*. found that elevation of transaminases and acute hepatic injury was independently related to worse outcomes in COVID-19 patients, including ICU admission, mechanical ventilation, and mortality in a large cohort of 1040 COVID-19 patients from Hong Kong [24]. Wu *et al*. reported a higher incidence of abnormal liver enzyme abnormality in non-survivors compared to survivors of COVID-19 (RR 1.34, P=0.04) [13]. The odds of mechanical ventilation and death in our cohort were higher in the liver involvement group. However, it did not reach statistical significance.

Several mechanisms for COVID-19-related hepatic damage have been proposed. Direct hepatic infection by SARS-CoV-2, which enters hepatocytes via ACE 2 receptors, is one probable reason for the liver injury reported in COVID-19 patients [25]. Post-mortem studies confirmed ultrastructural and histological evidence of typical viral infection of hepatocytes [26]. Another proposed mechanism is bile duct alteration, shown in a post-mortem analysis of 40 COVID-19 patients who developed liver injury. The most common observation was macrovesicular steatosis, followed by mild lobular necroinflammation, portal inflammation, and vascular pathology, including sinusoidal microthrombi. Additionally, 55% of patients had positive PCR of SARS-CoV2 from liver tissue [27]. Moreover, the activation of innate immune cells and the production of cytokines is a well-known cause of liver injury from various sources [28]. Likewise, patients with COVID-19 disease present an activation of inflammatory markers and cytokines [29]. Thus, deregulation of the immune system may be a possible mechanism.

Finally, we found that non-Saudi nationality and older age were associated with severe or critical COVID-19 pneumonia, as well as poor outcomes. DM increased the likelihood of severe or critical COVID-19 infection. Similarly, earlier research established a link between advanced age and an increased risk of ICU hospitalizations, intubations, and mortality [30]. Moreover, Aleanizy *et al*. reported that non-Saudi COVID-19 patients had a higher chance of severe to critical disease [31]. Possible explanations that could contribute to the worse outcomes in non-Saudi populations are genetic variations and the low socioeconomic status of such patients that contribute to their late presentation to hospitals. Similar to our findings, several studies found a link between DM and an increased risk of severe COVID-19 disease [32, 33].

Our study has two strong points. First, up to the date of writing this report, this was the first study conducted in Saudi Arabia and the Middle East investigating COVID-19 hepatic manifestations and their impact on the severity and outcome. Second, the data were collected systematically by a predetermined protocol to reduce extraction errors and missing data. However, being an observational research from a single center limits the results of our study. Future randomized controlled trials to support our results are still needed to improve the therapeutic outcome of COVID-19 patients.

## Conclusion

Liver involvement secondary to COVID-19 disease is a common clinical manifestation. Such patients are more susceptible to severe-critical disease and a worse clinical outcome in the form of longer hospitalization and greater need for ICU admission. It is of great importance to pay attention to hepatic manifestations in COVID-19 patients to categorize their disease severity and predict their outcome.

## References

[1] World Health Organization WHO Timeline – COVID-19. https://www.who.int/news/item/27-04-2020-who-timeline---covid-19.

[2] Coronavirus 2019nCov Statistics Update (Live). https://virusncov.com/.

[3] World Health Organization Saudi Arabia: WHO Coronavirus Disease (COVID-19) Dashboard. https://covid19.who.int/region/emro/country/sa.

[4] Guan W, Ni Z, Hu Y, Liang W, Ou C (2020). Clinical characteristics of Coronavirus Disease 2019 in China. New England Journal of Medicine.

[5] Huang C, Wang Y, Li X, Ren L (2020). Clinical features of patients infected with 2019 novel coronavirus in Wuhan, China. The Lancet.

[6] Shi H, Han X, Jiang N, Cao Y (2020). Radiological ndings from 81 patients with COVID-19 pneumonia in Wuhan, China: a descriptive study. Lancet Infect Dis.

[7] Chen T, Wu D, Chen H, Yan W (2020). Clinical characteristics of 113 deceased patients with coronavirus disease 2019: Retrospective Study. BMJ.

[8] Yang X, Yu Y, Xu J, Shu H (2020). Clinical course and outcomes of critically ill patients with SARS-COV-2 pneumonia in Wuhan, China: A single-centered, retrospective, Observational Study. The Lancet Respiratory Medicine.

[9] Ministry of Health MOH protocol for patients suspected/ or confirmed with COVID-19. https://www.moh.gov.sa/Ministry/MediaCenter/Publications/Documents/MOH-therapeutic-protocol-for-COVID-19.pdf.

[10] Henry BM, Aggarwal G, Wong J, Benoit S (2020). Lactate dehydrogenase levels predict coronavirus disease 2019 (COVID-19) severity and mortality: A pooled analysis. Am J Emerg Med.

[11] Cai Q, Huang D, Yu H, Zhu Z (2020). COVID-19: Abnormal liver function tests. J Hepatol.

[12] Zhang C, Shi L, Wang FS (2020). Liver injury in COVID-19: management and challenges. Lancet Gastroenterol Hepatol.

[13] Wu Y, Li H, Guo X, Yoshida EM (2020). Incidence, risk factors, and prognosis of abnormal liver biochemical tests in COVID-19 patients: a systematic review and meta-analysis. Hepatol Int.

[14] Wang Z, Yang B, Li Q, Wen L, Zhang R (2020). Clinical Features of 69 Cases With Coronavirus Disease 2019 in Wuhan, China. Clin Infect Dis.

[15] Richardson S, Hirsch JS, Narasimhan M, Crawford JM (2020). Presenting characteristics, comorbidities, and outcomes among 5700 patients hospitalized with covid-19 in the New York City area. JAMA.

[16] Lv Y, Zhao X, Wang Y, Zhu J (2021). Abnormal Liver Function Tests Were Associated With Adverse Clinical Outcomes: An Observational Cohort Study of 2,912 Patients With COVID-19. Front Med (Lausanne).

[17] Fan Z, Chen L, Li J, Cheng X (2020). Clinical Features of COVID-19-Related Liver Functional Abnormality. Clin Gastroenterol Hepatol.

[18] Zhang H, Liao YS, Gong J, Liu J, Zhang H (2020). Clinical characteristics and risk factors for liver injury in COVID-19 patients in Wuhan. World J Gastroenterol.

[19] Duan ZP, Chen Y, Zhang J, Zhao J (2003). [Clinical characteristics and mechanism of liver injury in patients with severe acute respiratory syndrome]. Zhonghua Gan Zang Bing Za Zhi.

[20] Li X, Xu S, Yu M, Wang K (2020). Risk factors for severity and mortality in adult covid-19 inpatients in Wuhan. Journal of Allergy and Clinical Immunology.

[21] Parohan M, Yaghoubi S, Seraji A (2020). Liver injury is associated with severe coronavirus disease 2019 (COVID-19) infection: A systematic review and meta-analysis of retrospective studies. Hepatol Res.

[22] Xu W, Huang C, Fei L, Li Q, Chen L (2021). Dynamic Changes in Liver Function Tests and Their Correlation with Illness Severity and Mortality in Patients with COVID-19: A Retrospective Cohort Study. Clinical Interventions in Aging.

[23] Wan S, Xiang Y, Fang W, Zheng Y (2020). Clinical features and treatment of COVID-19 patients in Northeast Chongqing. Journal of Medical Virology.

[24] Yip TC, Lui GC, Wong VW, Chow VCY (2020). Liver injury is independently associated with adverse clinical outcomes in patients with COVID-19. Gut.

[25] Hoffmann M, Kleine-Weber H, Schroeder S, Krüger N (2020). SARS-CoV-2 cell entry depends on ACE2 and TMPRSS2 and is blocked by a clinically proven protease inhibitor. Cell.

[26] Wang Y, Liu S, Liu H, Li W (2020). SARS-COV-2 infection of the liver directly contributes to hepatic impairment in patients with COVID-19. Journal of Hepatology.

[27] Lagana SM, Kudose S, Iuga AC, Lee MJ (2020). Hepatic pathology in patients dying of COVID-19: A series of 40 cases including clinical, histologic, and virologic data. Modern Pathology.

[28] McDonald B, Kubes P (2016). Innate immune cell trafficking and function during sterile inflammation of the liver. Gastroenterology.

[29] Liu F, Li L, Xu M, Wu J (2020). Prognostic value of interleukin-6, C-reactive protein, and procalcitonin in patients with COVID-19. J Clin Virol.

[30] Martos-Benítez FD, Soler-Morejón CD, García-del Barco D (2021). Chronic comorbidities and clinical outcomes in patients with and without COVID-19: a large population-based study using national administrative healthcare open data of Mexico. Intern Emerg Med.

[31] Aleanizy FS, Alqahtani FY, Alanazi MS, Mohamed RAEH (2021). Clinical characteristics and risk factors of patients with severe COVID-19 in Riyadh, Saudi Arabia: A retrospective study. J Infect Public Health.

[32] Li X, Xu S, Yu M, Wang K (2020). Risk factors for severity and mortality in adult COVID-19 inpatients in Wuhan. J Allergy Clin Immunol.

[33] Du Y, Tu L, Zhu P, Mu M (2020). Clinical Features of 85 Fatal Cases of COVID-19 from Wuhan. A Retrospective Observational Study. Am J Respir Crit Care Med.

